# LAD-III, a Mild Phenotype Resulting From a Novel Variant of FERMT3 Gene: A Case Report

**DOI:** 10.7759/cureus.51062

**Published:** 2023-12-25

**Authors:** Badriah G Alasmari, Mohammed Alomari, Wejdan N Alotaibi, Ashwaq Hommadi, Abdelhakam A Elmugadam, Khalid Abdalla, Saeed M Al-Tala

**Affiliations:** 1 Pediatrics, Armed Forces Hospital Southern Region, Khamis Mushait, SAU; 2 Pediatric Hematology Oncology, King Abdulaziz Medical City, Jeddah, SAU

**Keywords:** leukocyte adhesion deficiency, whole exome sequencing (wes), ecchymosis, fermt3 gene, primary immune deficiency

## Abstract

Leukocyte adhesion deficiency-III (LAD-III) is a rare recessive autosomal disorder characterized by bleeding syndrome of Glanzmann-type and life-threatening infections. The main etiology of this condition is variations in the *FERMT3* gene, which encodes kindlin-3, an integrin-binding protein. This protein is responsible for the activation of fibrinogen receptors and integrin-mediated hematopoietic cell adhesion. So far, only limited cases of LAD-III have been reported. This case report discusses a two-year-old male infant from the Asir region, Saudi Arabia, who was referred to the pediatric hematology service due to recurrent ecchymosis and epistaxis. He was born at full term with a history of transient tachypnea of the newborn and recurrent bronchiolitis. The patient exhibited normal platelet count and coagulation profiles alongside a familial history of bleeding disorders, including a cousin with a similar condition. The patient also presented with hypospadias and café-au-lait spots. Laboratory findings revealed anemia, microcytosis, and hypochromia indicative of iron deficiency anemia. Whole exome sequencing (WES) identified a homozygous variant of uncertain significance in the *FERMT3* gene, associated with autosomal recessive LAD-III. The patient was subsequently referred to an immunology subspecialty for further investigation and bone marrow transplant preparation. This case underscores the importance of comprehensive clinical and genetic evaluations in pediatric patients with unexplained bleeding tendencies.

## Introduction

Leukocyte adhesion defects (LADs) constitute a rare group of primary immunodeficiency disorders that lead to impairments in molecules crucial for the adhesion cascade during leukocyte mobilization to inflammatory sites [1]. LADs are classified into types I, II, and III, characterized by homozygous pathogenic variants in the *ITGB2*, *SLC35C1*, and *FERMT3* genes, respectively [2]. Recently, a novel LAD type IV has also been described which is characterized by pathogenic variants in the transmembrane conductance regulator (*CFTR*) gene [3]. *FERMT3* genes encode for kindlin-3, a protein involved in the activation of integrin which ensures the adhesion of platelets, granulocytes, and lymphocytes. In LAD-III, there is a variation of the *FERMT3* gene that impairs integrin function [4]. LAD-III manifests as abnormal integrin activation during the second phase of the adhesion cascade [5]. Clinical symptoms in LAD-III patients include severe recurrent infections, a propensity for bleeding, and pronounced leukocytosis [6]. The bleeding episodes are primarily caused by platelet aggregation dysfunction. Furthermore, some individuals may exhibit bone defects reminiscent of osteopetrosis. The management of LAD-III involves frequent blood transfusions and antibacterial therapy for infections. However, bone marrow transplantation is the sole curative therapy [7]. In this case report, we describe a case of LAD-III phenotype caused by a novel variant of the *FERMT3 *gene.

## Case presentation

A two-year-old male infant was transferred from general pediatrics to the pediatric hematology service due to a history of recurrent ecchymosis. These symptoms were first noted by his mother at the age of 10 months. The child was born at full term to consanguineous parents from the Asir region, Saudi Arabia. The patient had a brief neonatal intensive care unit (NICU) admission due to transient tachypnea of the newborn. Furthermore, the patient had recurrent episodes of bronchiolitis. There were a few episodes of epistaxis and the separation of the cord stump at 12 days of age. No blood diarrhea, bloody vomiting, joint swelling, or changes in mental status were reported.

**Figure 1:**
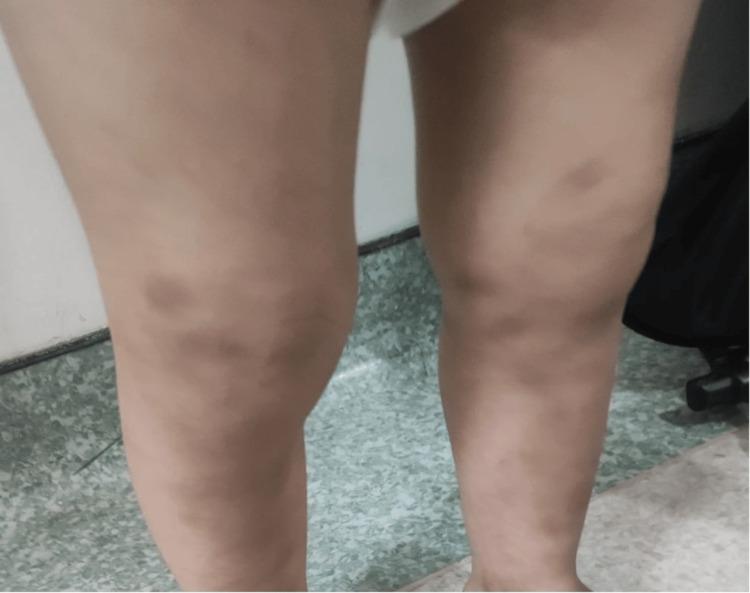
Ecchymosis of the lower limb.

The mother of the patient noticed that the recurrent episodes of ecchymosis in his lower legs (Figure 1) disappeared on their own. The patient's cousin also had a similar condition. Upon examination, all findings were unremarkable, except for café-au-lait spots, ecchymosis in the lower limbs, and hypospadias. Laboratory findings are given in Table 1.

**Table 1:** Complete blood count (CBC) of patient.

Description	Result	Reference Range
White Blood Cells (WBCs)	26.5 x 10^9^/L	5-15.5
Red Blood Cells (RBCs)	4.00 x 10^12^/L	3.8-5.2
Hemoglobin	9.0 g/dL	9.2-13.5
Hematocrit (HCT)	30.6%	31-41
Mean Corpuscular Volume (MCV)	76.6 fL	70-86
Mean Corpuscular Hemoglobin (MCH)	22.6 pg	23-30
Mean Corpuscular Hemoglobin Concentration (MCHC)	29.5 g/dL	32-36
Red Cell Distribution Width (RDW)	19.5%	11-14
Platelet Count (PLT)	336 x 10^9^/L	150–450
Mean Platelet Volume (MPV)	10.2 fL	9.4-12.3
Absolute Lymphocyte (LYMPH ABS)	17.59 x 10^9^/L	4-10.5
Absolute Large Unstained Cells (LUC ABS)	1.43 x 10^9^/L	0-0.4

**Figure 2:**
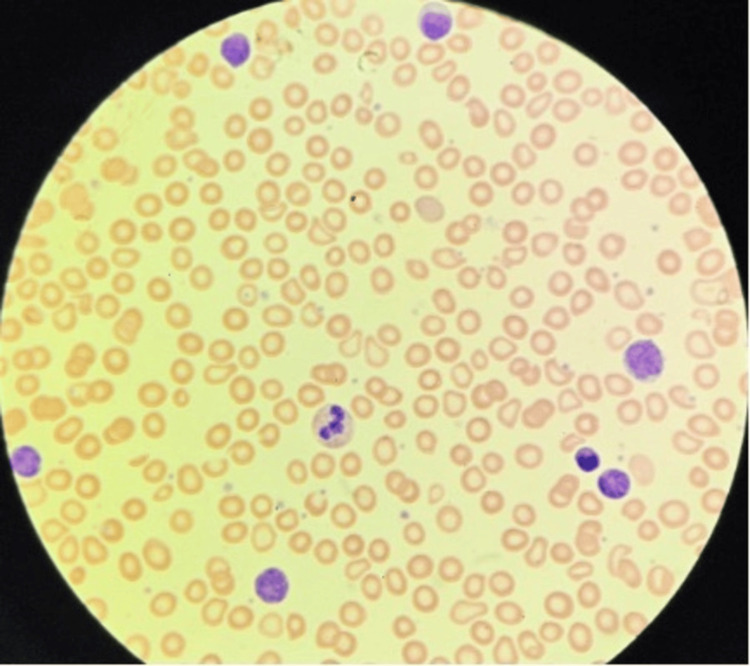
Peripheral blood smear showing lymphocytosis.

Peripheral blood analysis showed hypochromic microcytic and occasional polychromasia with Rouleux formation, consistent with iron deficiency anemia. WBC analysis revealed lymphocytosis (75%), with occasionally activated forms requiring verification for reactive causes (Figure 2).

The eosinophil count was 5%. The platelet and neutrophil counts were adequate. No classical blasts were observed. Furthermore, a platelet function assay was performed and it was prolonged.

**Figure 3:**
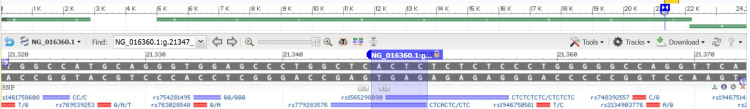
The genomic viewer photo clip of the variant genomic sequence position.

Whole-exome sequencing (WES) identified a homozygous variant (c.1683-22_1683-19del) of uncertain significance in the *FERMT3* gene (class 3) according to CENTOGENE® (Centogene, Rostock, Germany) and the American College of Medical Genetics (ACMG). The variant c.1683-22_1683-19del p. is a deletion mutation that removes four nucleotides from the DNA sequence of the *FERMT3* gene. This mutation affects the splicing of the gene, The mutation is predicted to cause the splicing to skip an exon, and result in an abnormal protein product if it escapes nonsense-mediated decay. The abnormal protein product function is predicted to be affected, leading to the LAD-III phenotypes (Figure 3).

According to the Verismo database, this variant is novel and not present in the clinVar database. Pathogenic variants in this gene are associated with autosomal recessive leukocyte adhesion deficiency type III. The patient was subsequently referred to the immunology subspecialty for further investigations and bone marrow transplant preparation.

## Discussion

This case study presents a two-year-old male infant with a history of recurrent ecchymosis, bleeding episodes, and respiratory issues, ultimately diagnosed with LAD-III. In the present study, we identified a novel homozygous variant (c.1683-22_1683-19del) of uncertain significance in the *FERMT3* gene. This variant is classified as a variant of unknown significance (class 3). LAD-III was first described in 1997 [8]. Since then, approximately 40 cases of LAD-III have been reported in the literature [9]. According to GnomAD v2.1.1, there are 1,112 variations in FERMT3 [10]. Shahid et al., in their study, reported a novel homozygous, stop codon variant c.C286T (p.Q96∗) in the *FERMT3* gene in a Pakistani family [11]. Similarly, Wolach et al. reported c.1069C > T in *FERMT3* in LAD-III patients [12]. They further reported a survival rate of 40% in LAD-III patients who had successful hematopoietic stem cell transplantation.

Features of the patient’s phenotype include umbilical cord separation at 12 days of age, which is higher than standard cord separation time (reference mean ± SD time = 6.61 ± 2.33 days) [13]. In the present case, the clinical history of the patient is recurrent ecchymosis, bleeding episodes, and recurrent episodes of bronchiolitis. This aligns with the commonly reported findings of LAD-III patients. Previously, Wolach et al. reported that 72% of infections in LAD patients were in the skin region [12]. Skin lesions in LAD are not characterized by pus as it has impaired leukocyte mobilization in inflammatory sites. In the present case, the patient had mild gum bleeding and episodes of epistaxis. These recurrent manifestations can be attributed to abnormality in integrin function. Integrins play an important part in the formation of new stromal layers by synthesizing new matrix and stimulating growth factors. Furthermore, they are also involved in forming granulation tissues, angiogenesis, and proliferation of fibroblasts which leads to gingival reepithelization and reconstitution of basal membrane [14,15]. 

In the current case, recurrent episodes of epistaxis and ecchymosis were the most prominent clinical manifestations that triggered genetic evaluation. Diagnostic exome sequencing is the go-to approach for detecting 50% of inborn errors in metabolism [16]. A key feature of the current case is lymphocytosis (75%), with occasionally activated forms. This is consistent with previous findings from a LAD-III case presented by Yahya et al. who reported persistent lymphocytosis and agranulocytosis [4]. Leukocytosis was quite prominent in the present case (26.5x10^9^/L). The differential diagnosis of leukocytosis in LAD-III includes infections, leukemoid reactions, chronic myelogenous leukemia, and lymphoproliferation [17]. However, LAD-III can be differentiated based on increased lymphocytes [18]. Generally, LAD-III should be differentiated from LAD-I, leukemoid reaction, and Glanzmann thrombasthenia.

The management of LAD-III includes supportive treatment including topical thrombin and anti-fibrinolytic agents (aminocaproic acid). In cases of severe bleeding, platelet transfusion is also recommended. Furthermore, skin and mucosal hygiene is necessary in LAD-III patients. Antimicrobial therapy is necessary in case of infection [19]. Bone marrow transplant is an effective therapy in patients with LAD-III. A case report by Jurk et al. reported that following allogeneic hematopoietic stem cell transplantation, the patient showed normalization of platelet, granulocyte functions, and osteopetrosis [18].

## Conclusions

This case study highlights the complexity of diagnosing rare immunodeficiency disorders like LAD-III. The integration of clinical, genetic, and hematological findings is paramount for accurate diagnosis and appropriate management. Our patient presented with symptoms of recurrent ecchymosis, gum bleeding, epistaxis, and ecchymosis in the lower limbs. These findings instigated whole exome sequencing and identified a homozygous variant of uncertain significance in the *FERMT3* gene, associated with autosomal recessive LAD-III. The patient had leukocytosis and lymphocytosis. The patient was referred to immunology for further evaluation and bone marrow transplant preparation. As our understanding of LAD-III continues to evolve, the identification of novel genetic variants and expanded clinical phenotypes contributes to the collective knowledge that guides future research and clinical practice.
